# RNA Interference in the Tobacco Hornworm, *Manduca sexta*, Using Plastid-Encoded Long Double-Stranded RNA

**DOI:** 10.3389/fpls.2019.00313

**Published:** 2019-03-14

**Authors:** William G. Burke, Emine Kaplanoglu, Igor Kolotilin, Rima Menassa, Cam Donly

**Affiliations:** ^1^London Research and Development Centre, Agriculture and Agri-Food Canada, London, ON, Canada; ^2^Department of Biology, The University of Western Ontario, London, ON, Canada; ^3^Scattered Gold Biotechnology Inc., London, ON, Canada

**Keywords:** RNA interference, *Manduca sexta*, tobacco hornworm, *Nicotiana tabacum*, plastid transformation, *v-ATPaseA*, long dsRNA

## Abstract

RNA interference (RNAi) is a promising method for controlling pest insects by silencing the expression of vital insect genes to interfere with development and physiology; however, certain insect Orders are resistant to this process. In this study, we set out to test the ability of *in planta*-expressed dsRNA synthesized within the plastids to silence gene expression in an insect recalcitrant to RNAi, the lepidopteran species, *Manduca sexta* (tobacco hornworm). Using the *Manduca vacuolar-type H^+^ ATPase subunit A* (*v-ATPaseA*) gene as the target, we first evaluated RNAi efficiency of two dsRNA products of different lengths by directly feeding the *in vitro*-synthesized dsRNAs to *M. sexta* larvae. We found that a long dsRNA of 2222 bp was the most effective in inducing lethality and silencing the *v-ATPaseA* gene, when delivered orally in a water droplet. We further transformed the plastid genome of the *M. sexta* host plant, *Nicotiana tabacum*, to produce this long dsRNA in its plastids and performed bioassays with *M. sexta* larvae on the transplastomic plants. In the tested insects, the plastid-derived dsRNA had no effect on larval survival and no statistically significant effect on expression of the *v-ATPaseA* gene was observed. Comparison of the absolute quantities of the dsRNA present in transplastomic leaf tissue for *v-ATPaseA* and a control gene, *GFP*, of a shorter size, revealed a lower concentration for the long dsRNA product compared to the short control product. We suggest that stability and length of the dsRNA may have influenced the quantities produced in the plastids, resulting in inefficient RNAi in the tested insects. Our results imply that many factors dictate the effectiveness of *in planta* RNAi, including a likely trade-off effect as increasing the dsRNA product length may be countered by a reduction in the amount of dsRNA produced and accumulated in the plastids.

## Introduction

Utilization of the RNA interference (RNAi) pathway, first discovered in *Caenorhabditis elegans* (Fire et al., 1998) and later observed in a wide variety of species (Bellés, 2010), to knockdown gene expression is a popular tool for biological research involving insects (Dietzl et al., 2007). Initially used to observe phenotypic effects of gene knockdowns, using RNAi knockdown of vital genes to induce a lethal response has been suggested as a means for developing species-specific pest control methods that are alternatives to potentially harmful chemical methods (Whyard et al., 2009). In insects, the RNAi pathway can be activated through environmental RNAi, whereby exogenous double-stranded RNA (dsRNA) molecules taken up from the environment, trigger the post-transcriptional silencing of endogenous mRNA molecules (Whangbo and Hunter, 2008). This process is accomplished by uptake of ingested dsRNA molecules from the midgut lumen into the insect cells through SID-1 channels and receptor-mediated endocytosis (Shih and Hunter, 2011; Cappelle et al., 2016). Later, the dsRNA molecules are processed into small interfering RNA (siRNA) molecules that induce gene knockdown (Meister and Tuschl, 2004). Thus, oral consumption is considered an effective tool for introducing dsRNA into insect cells for developing RNAi-based pest control strategies.

The dsRNA to be delivered to insects is commonly synthesized *in vitro* or expressed in bacteria (Timmons et al., 2001; Flores-Escobar et al., 2013). However, a more useful strategy for application in a greenhouse or field setting is *in planta* expression by the crop itself. Nuclear dsRNA expression was initially tested and has been successful for pest insects such as western corn rootworm (Baum et al., 2007). Yet, success for insects within the crop-devastating clade Lepidoptera is generally less pronounced, and complete plant resistance to feeding through nuclear dsRNA expression has not been achieved (Terenius et al., 2011). Plants, like other eukaryotes, possess an innate RNAi pathway (Melnyk et al., 2011), and thus process dsRNAs into siRNA prior to insect ingestion. In fact, gene knockout of RNAi processing pathway components within nuclear dsRNA-expressing plants increases the ability of these plants to resist feeding by cotton bollworm (Mao et al., 2007), a result which suggests that the plant’s intrinsic ability to process dsRNA into siRNA is responsible for the reduced RNAi efficiency in insects, because siRNA uptake in the insect midgut lumen is less efficient than long dsRNA uptake (Bolognesi et al., 2012).

Expression of dsRNA within the plastid presents a possible solution for overcoming this problem. Plastids are prokaryotic in origin and do not maintain an RNAi pathway, thus dsRNA should remain intact prior to insect consumption. This strategy was successful in knocking down gene expression in Colorado potato beetle (Zhang et al., 2015) and cotton bollworm (Jin et al., 2015; Bally et al., 2016). These results indicate that this strategy could potentially prove effective in imparting *in planta* protection from insect herbivory, but the full range of properties that influence success are yet to be elucidated. For instance, dsRNA molecule size and sequence play a role in effectiveness of the approach and must be examined individually for any specific research goal. With respect to molecule size, longer dsRNA products are generally associated with greater uptake and utilization by the insect RNAi pathway (Miller et al., 2012). Previously, a study comparing the effectiveness of *in vitro* synthesized long dsRNA and an artificial microRNA (miRNA) targeting *chitin synthase gene A* in the brown planthopper revealed that long dsRNA was more effective in gene silencing, resulting in more significant effects on fecundity and ovary development compared to miRNA (Li et al., 2017). Also, although several other studies using *in planta* expressed miRNA-based approaches showed positive results (Agrawal et al., 2015; Jiang et al., 2017; He et al., 2019), complete plant resistance to insect herbivory was not accomplished in most cases. With regard to molecule sequence, the range of insect species affected by a single dsRNA product depends on the gene target. For instance, dsRNAs targeting western corn rootworm *vacuolar-type H^+^ ATPase subunits A* and *E* (*v-ATPaseA* and *v-ATPaseE*) genes cause significant mortality in Colorado potato beetle (Baum et al., 2007). Similarly, dsRNAs that target two midgut genes in tobacco hornworm, *Manduca sexta*, silence homologous genes in tomato hornworm (Poreddy et al., 2017). These results imply that dsRNA molecules complementary to conserved genes offer a greater range of possible insect targets, which could be beneficial for field application.

In this study, we set out to evaluate the effect of dsRNA length on RNAi efficiency by first comparing the effectiveness of two *in vitro*-synthesized dsRNA molecules of different sizes (long, 2222 bp and short, 259 bp) at inducing gene silencing and mortality in larvae of the tobacco hornworm, *M. sexta*, a widely used insect model in molecular biology. The dsRNAs target the *v-ATPaseA* gene, which has been demonstrated to be an effective target for achieving lethality in numerous insect species (Baum et al., 2007; Ulrich et al., 2015; Yu et al., 2016). Then, we transformed the plastid genome of tobacco, *Nicotiana tabacum*, with an expression cassette producing the long *v-ATPAseA* dsRNA, as this fragment was the most effective at inducing RNAi when fed as *in vitro*-synthesized products. This product is larger than dsRNAs produced in chloroplasts in previous studies (Jin et al., 2015; Zhang et al., 2015; Bally et al., 2016). Bioassays with *M. sexta* larvae were then performed on the transplastomic plants to determine whether *in planta*-expressed dsRNA could induce lethality in feeding insects. Finally, we quantified expression of the long dsRNA product and compared with expression of a shorter 223 bp dsRNA product to determine whether the size of the dsRNA had any effect on the amount of dsRNA produced in the plastids.

## Materials and Methods

### Tobacco Growth and Variety

The tobacco plants used in this study were of the variety “81V9,” a low-alkaloid cultivar (Menassa et al., 2001). Tobacco seeds were sterilized by a 5-min wash with 70% ethanol and grown in Magenta vessels containing Murashige and Skoog (MS) medium (4.4 g/L Murashige and Skoog Basal Medium with Vitamins, 3% sucrose and 0.7% agar). Plants for bioassays were transferred to soil and grown under 16L:8D light cycles in greenhouses under ambient conditions. All tissue culture stages were performed at room temperature under a 16L:8D photoperiod.

### *In vitro* dsRNA Synthesis

PCR primers (Supplementary Table 1) were designed to amplify product sizes of 2222 bp (*v-ATPAseA* long) and 259 bp (*v-ATPaseA* short) from *M. sexta* cDNA. The resulting dsRNA sequences are listed in Supplementary Table 2. The primers were designed such that *v-ATPAseA* short was contained within *v-ATPAseA* long (Supplementary Figure 1) and sequence identity with homologs in other organisms is shown in Supplementary Figure 2. A 223 bp product of *green fluorescent protein* (*GFP*) from *Aequorea victoria* was used as a dsRNA control. All primers contained T7 RNA polymerase promoter sequences at their 5′ end for *in vitro* dsRNA synthesis. Templates for *in vitro* dsRNA synthesis were prepared by PCR amplification, followed by gel purification using the QIAquick Gel Extraction Kit (Qiagen). Double-stranded RNA targeting *v-ATPaseA* was synthesized using the MEGAscript T7 Transcription Kit (Ambion) while dsRNA targeting *GFP* was synthesized using the RiboMAX Express RNAi System (Promega) following the manufacturer’s instructions.

### Construction of the Plastome Transformation Vector (pTomCT)

With a long-term goal of creating pest-resistant tomato plants via plastome engineering for dsRNA production, we designed and constructed a tomato plastome transformation vector pTomCT. Given the prolonged reported timeframe for generating transplastomic tomato plants (Ruf et al., 2001) and the existence of much shorter and relatively routine procedures for tobacco plastid transformation, we initially tested the utility of the pTomCT vector by transforming tobacco chloroplasts. We hypothesized that the high level of homology between tomato and tobacco plastomes (99% homology in the targeted region) would facilitate efficient transformation and regeneration of transplastomic clones in tobacco.

**FIGURE 1:**
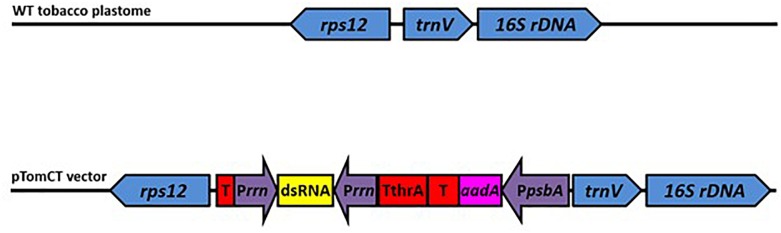
Schematic representation of the tobacco plastome integration site (top) and the transformation construct pTomCT (bottom). Blue: *rps12*-*trnV*-*16s rDNA* integration site; Red: Transcription terminators (T), including the threonine attenuator (TthrA); Yellow: Region where sequences from *Manduca sexta v-ATPaseA* and *GFP* genes were cloned for dsRNA production; Purple: promoters including *PpsbA* – *psbA* gene promoter for *aadA* gene expression, *Prrn* – chloroplast *rrn* operon promoter; Pink: *aadA* – gene encoding aminoglycoside 3′ adenylyltransferase for spectinomycin resistance.

The tomato plastome transformation construct pTomCT was designed to insert/integrate the expression cassette into a transcriptionally silent intergenic spacer between the *rps12* and *trnV* genes of the tomato plastome NC_007898 (Figure 1), thus eliminating potential interference from endogenous transcriptional activity. For this purpose, DNA sequences between nucleotides 99,339 – 100,883 and 100,884 – 102,396 of the tomato plastome were used as the left and right flanking sequences of the expression cassette, respectively. The synthetic DNA comprising the flanking regions and the expression cassette was directionally cloned into *Kas* I and *Hind* III restriction sites on the pUC57 (AMP^R^) plasmid, which served as the backbone vector.

The expression cassette (Figure 1) consists of two *cis*-linked expression modules, separated by an efficient transcriptional terminator of the *Escherichia coli* threonine attenuator (*TThrA*) (Gardner, 1982; Chen and Orozco, 1988), designed to disjoin the transcriptional modules in the expression cassette. The first transcriptional module in the cassette was designed for expression of a selective marker and contains the core promoter and the 5′-untranslated region of the plastid *psbA* gene (*PpsbA*), linked to the *aadA* gene open reading frame with a stop codon, followed by a heterologous untranslated sequence found on the 3′ end of the *psbC* gene of the *Populus alba* plastome (Kolotilin et al., 2013). The second transcriptional module contains two inward facing *rrn* operon promoters (*Prrn*) with a multiple cloning site between them (Zhang et al., 2015). Both promoters were engineered with two triplet nucleotide mutations (“-58 - -56” ATG to TAC; and “-22 – -20” AGG to TCC) (Suzuki et al., 2003) in order to reduce the homology to the endogenous *Prrn*, thus reducing the probability of deleterious homologous recombination. Upstream of the endogenous *rps12* gene a heterologous untranslated DNA sequence (T2) found at the 3′-end of the *rbcL* gene of the *P. alba* plastome (Kolotilin et al., 2013) was introduced in order to eliminate possible transcriptional interference from the expression cassette.

To clone dsRNA encoding sequences, primers containing *Not* I and *Sal* I restriction sites at their 5′-ends were used to generate PCR products, which were then ligated into the multi-cloning site between the two convergent *Prrn* promoters on the expression cassette. Two dsRNA fragments [*v-ATPAseA* (2222 bp) and *GFP* (223 bp)] were cloned into pTomCT for plastid transformation. Primers used for cloning of the fragments are listed in Supplementary Table 1.

### Generation of Transplastomic Tobacco Lines

Plastid transformation was accomplished via the biolistic method (Daniell et al., 2005; Verma et al., 2008). After bombardment, leaves were cut into approximately 1 cm^2^ pieces and distributed onto petri dishes containing regeneration medium (MS medium containing 500 mg/L spectinomycin, 1 mg/L 6-benzylaminopurine, 0.1 mg/L 1-naphthaleneacetic acid, 1 mg/L thiamine hydrochloride, and 0.1 g/L myo-inositol). Potential transformants were detected by the growth of small, green shoots from the explants. Shoots were cut and returned to regeneration medium three times to ensure homoplastomy of the transgenic lines. Following the third round of selection, tissue was placed on MS medium with 500 mg/L spectinomycin for induction of root growth. Following the appearance of roots, plants were moved to soil for further growth.

### RNA and DNA Extraction

Tissue samples from tobacco leaves were frozen in liquid nitrogen and disrupted using a TissueLyser II (QIAGEN). Total DNA was extracted using the CTAB DNA extraction protocol (Porebski et al., 1997), while total RNA was extracted using an acid phenol:chloroform extraction method (Deepa et al., 2014). To extract RNA from insects, RNeasy Mini Kit (QIAGEN) was used following the manufacturer’s protocol. All RNA samples were treated with Turbo RNase-Free DNase (Ambion) to prevent genomic DNA contamination in subsequent steps.

### Southern Blotting to Confirm Homoplastomy of the Transgenic Tobacco

Southern blotting was performed as described previously (Daniell et al., 2005; Southern, 2006). Briefly, tobacco DNA was digested using restriction enzymes *Pfo* I and *Nde* I, which cut regions within the flanking sequences used for homologous recombination. The hybridization probe was prepared using the PCR DIG Probe Synthesis Kit (Sigma-Aldrich) with primers (Supplementary Table 1) that generated a 1181 bp DIG-labeled probe from the homologous recombination regions. Digested DNA was separated on a 0.8% w/v agarose gel and transferred overnight to an Amersham Hybond membrane (GE Healthcare). The membrane was crosslinked at 1200V with a CL-1000 Crosslinker (UVP) and hybridized with the probe overnight at 50°C in DIG Easy Hyb buffer (Roche). After two washes in 2 × Saline Sodium Citrate (SSC, 20 × SSC is 3.0 M NaCl, 0.3 M sodium citrate, pH = 7.0) buffer + 0.1% SDS at room temperature and three washes in 0.5 × SSC + 0.1% SDS at 68°C, membrane was blocked with DIG blocking buffer (Roche). Hybridized probe was detected by soaking membrane in Anti-Digoxigenin-AP Fab antibody solution (Roche) followed by CSPD chemiluminescent substrate (Roche) detection. A MicroChemi 4.2 Bio Imaging System was used to visualize the bands after one hr exposure.

### Confirmation of dsRNA Expression in Transplastomic Plants

To detect dsRNA expression, cDNA was synthesized from 500 ng total RNA using a Superscript III First-Strand Supermix Kit (Invitrogen) with a minor modification to the manufacturer’s protocol. Following the addition of 50 ng random hexamers, RNA was incubated at 95°C for 5 min to denature dsRNA molecules, and then immediately transferred to ice. Subsequent steps followed the manufacturer’s protocol. No reverse transcriptase controls (NRT) were also performed to confirm absence of genomic DNA carryover. PCR was performed using primers specific for *v-ATPAseA* or *GFP* dsRNA (Supplementary Table 1) using Taq DNA polymerase followed by agarose gel electrophoresis to visualize the products.

To demonstrate that dsRNA molecules are formed after transcription from the convergent *Prrn* promoters, 2 μg of total RNA from wild type (WT), *v-ATPAseA* and *GFP* dsRNA-producing plants was digested with RNAseA (Promega) to remove all single-stranded RNA (ssRNA). The reaction mixtures were incubated at 37°C for 30 min, and dsRNA was precipitated using isopropanol following the protocol from the RiboMAX Express RNAi System (Promega). The dsRNA pellets were then dissolved in 15 μL nuclease-free water and 7 μL of the digested RNA was used for cDNA synthesis, followed by PCR amplification, as described above.

### Droplet Digital PCR (ddPCR) to Quantify dsRNA Production in Transgenic *N. tabacum* Lines

To quantify dsRNA expression, cDNA was synthesized from 500 ng RNA as described in the previous section and used as template. PCR was performed using Bio-Rad ddPCR Supermix for probes, forward and reverse primers at 900 nM, and probes at 250 nM in a 20 μL final reaction volume. NRT controls were also run to ensure that templates were free of DNA contamination. Droplets were generated using the Bio-Rad QX100 Droplet Generator, followed by cycling on a Bio-Rad T100 Thermocycler with the following temperature profile: initial denaturation at 95°C for 10 min, 40 cycles of 94°C for 30 s, 60°C for 1 min, and a final incubation at 98°C for 10 min. Droplets were read on a Bio-Rad QX100 Droplet reader and data was analyzed using Quantasoft 1.7.4 software. Absolute quantification of dsRNA was done using *N. tabacum ribosomal protein L25* (*RPL25*) as a reference gene (Schmidt and Delaney, 2010). Quantities of dsRNA present in 500 ng total RNA were calculated using the guidelines from droplet digital PCR application guide (Bio-Rad, 2018). Statistical analysis of any differences between *v-ATPaseA* and *GFP* dsRNA levels was performed using a *t*-test.

### *M. sexta* Bioassays

Insect eggs and artificial diet were obtained from Reptile Feeders (Norwood, ON, Canada). The eggs were hatched on artificial diet for droplet feeding assays, and on WT tobacco leaves for *in planta* feeding assays. Insects were reared at 27°C and a 16L:8D light cycle. For droplet feeding assays, insects were first raised to second instar. Then, larvae were starved for 4–6 h and each presented with a 1 μL liquid droplet containing 2.5 μg of long or short *v-ATPaseA* dsRNA, *GFP* dsRNA, or water only. Any insect that did not consume the entire droplet was discarded from the bioassay. Insects were then moved to cups containing artificial diet and allowed to feed *ad libitum*. Survival was recorded daily and observed over a 7-day period. Additional insects were droplet-fed for RNA extraction after 3 days to confirm gene knockdown. For *in planta* feeding bioassays, first instar insects less than 24 h old were moved to cups containing 1% agarose and 3 cm diameter leaf disks from either WT, *v-ATPAse*A dsRNA-, or *GFP* dsRNA-expressing plants. Leaf disks were replaced daily and survival was recorded over a 7-day period. Survival of insects was compared using a Kaplan–Meier survival analysis and compared between treatments using log-rank tests.

### Gene Expression in *M. sexta* Larvae

To measure insect gene expression, cDNA was synthesized from 500 ng insect RNA using the SuperScript III First-Strand Synthesis SuperMix for RT-qPCR kit (Invitrogen) according to the manufacturer’s protocol. Reverse transcription quantitative PCR was performed using a SensiFAST SYBR No-ROX Mix Kit (Bioline) and a CFX96 Real-Time Detection System (Bio-Rad). Primers for RT-qPCR were designed to amplify an approximately 100 bp region of the *M. sexta v-ATPaseA* gene in a region outside of the dsRNA product to avoid spurious signal attributed to dsRNA carryover during extractions. Primers targeting the *M. sexta elongation factor 1-alpha* (*EF1-α*) gene were used as a reference (Supplementary Table 1). Gene expression knockdown was assessed between insect RNA samples using one-way ANOVA followed by Dunnett’s test.

## Results

### Knockdown of *M. sexta v-ATPaseA* Affects Larval Survival

**FIGURE 2:**
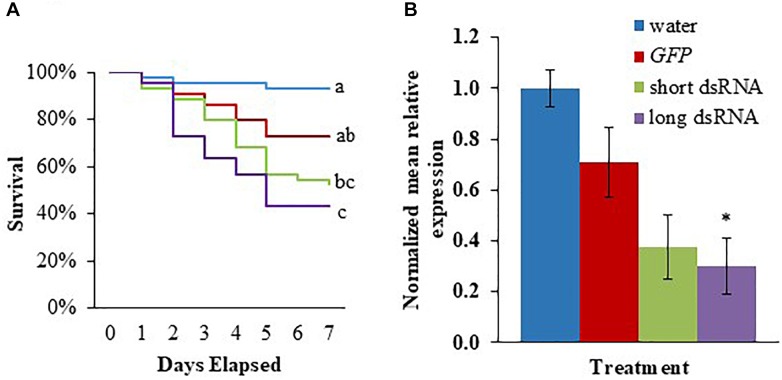
Survival of *M. sexta* second instar larvae and knockdown of *v-ATPaseA* gene after feeding with *in vitro* synthesized dsRNA. **(A)** Kaplan–Meier survival analysis over a 7-day period. Different letters represent significant difference according to log-rank tests (*p* < 0.05, *n* = 44), which were performed between each group. **(B)** Knockdown of *v-ATPaseA* gene in insects 3 days after feeding on dsRNA. Normalized mRNA quantity was set to one in water-fed larvae and relative differences in mRNA levels of treatment groups were calculated. Data are expressed as mean relative quantity ± SEM. Asterisks indicate significant difference from water control according to Dunnett’s test (^∗^*p* < 0.05, *n* = 4).

To determine whether *v-ATPaseA* dsRNA can induce gene knockdown and lethality in second instar *M. sexta* larvae, we fed the larvae a 1 μL water droplet containing 2.5 μg of *in vitro*-synthesized dsRNA: *v-ATPaseA* dsRNA long, *v-ATPaseA* dsRNA short, *GFP* dsRNA, or no dsRNA. After 7 days, 56.8% of insects fed the long *v*-*ATPaseA* dsRNA died, compared to 6.8% (log rank χ^2^ = 17.1, df = 1, *p* = 0.000035, *n* = 44) and 27.2% (log rank χ^2^ = 5.46, df = 1, *p* = 0.019, *n* = 44) for insects fed water and *GFP* dsRNA, respectively (Figure 2A). In larvae fed *v-ATPAseA* dsRNA short, mortality was 47.8%, which was lower than that for the long dsRNA product, but not significantly different (*p* > 0.05). Mortality in *v-ATPaseA* short-fed larvae was also significantly higher in comparison to water-fed larvae (log rank χ^2^ = 11.12, df = 1, *p* = 0.00085, *n* = 44); however, it was not significantly different from the *GFP* dsRNA-fed larvae (*p* > 0.05) (Figure 2A). To ensure that knockdown of *v-ATPaseA* mRNA was taking place, additional insects were treated in the same way and RNA was extracted after 3 days. Reverse transcription-qPCR indicated that *v-ATPaseA* expression was reduced significantly, by 68.9%, in insects fed long *v-ATPaseA* dsRNA product compared to insects fed water (*F* = 3.70, *p* = 0.042) (Figure 2B). Relative *v-ATPaseA* gene expression in larvae fed *v-ATPaseA* dsRNA short or *GFP* dsRNA compared to insects fed water was reduced 62.5 and 29.1%, respectively, which were not significantly different from water (*p* > 0.05).

### Transplastomic Plants Express *vATPase* and *GFP* dsRNA

**FIGURE 3:**
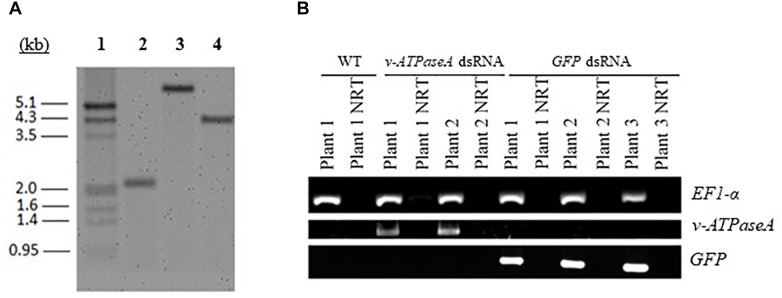
Characterization of transplastomic tobacco plants. **(A)** Southern blot confirming homoplastomy of the transformed tobacco lines. Tobacco DNA was cut with restriction enzymes and separated on an agarose gel for blotting. A probe hybridizing to homologous recombination regions in tobacco plastids was used to confirm insertion/integration of dsRNA expression cassettes into plastid DNA. A single band of the expected size was observed in each DNA sample; 1, DIG-labeled marker; 2 DNA from WT tobacco; 3, DNA from tobacco with *v-ATPaseA* dsRNA expression cassette; 4, DNA from tobacco with *GFP* dsRNA expression cassette. All DNA samples display single bands which confirm homoplastomy of transformed plants. **(B)** RT-PCR confirming expression of *v-ATPaseA* dsRNA and *GFP* dsRNA in transplastomic plants. Primers annealing to dsRNA sequences were used to confirm production of dsRNA in transplastomic tobacco plants. Tobacco *EF1-α* gene was used as a positive control and NRT reactions confirmed absence of DNA contamination in cDNA preparations.

Based on the results of the droplet feeding assays, the long *v-ATPaseA* fragment and the *GFP* control fragment were selected for expression in transplastomic plants. Following biolistic delivery of gold particles carrying the appropriate recombinant pTomCT vectors, leaf explants were placed on regeneration medium containing spectinomycin for the selection of transformants. Three rounds of selective regeneration were performed to ensure homoplastomy of the transgenic plants, which was confirmed using a Southern blot (Figure 3A). In WT tobacco, the expected restriction fragment size was 2102 bp while in transplastomic plants, they were 6183 and 4184 bp for *v-ATPaseA* and *GFP* dsRNA expression cassettes, respectively. Both WT and transplastomic tobacco plants displayed single bands of the expected sizes, and absence of the WT band in transplastomic plant lines confirmed homoplastomy. The resulting transplastomic plants showed no significant observable differences in their phenotype compared to WT plants (Supplementary Figure 3).

**FIGURE 4:**
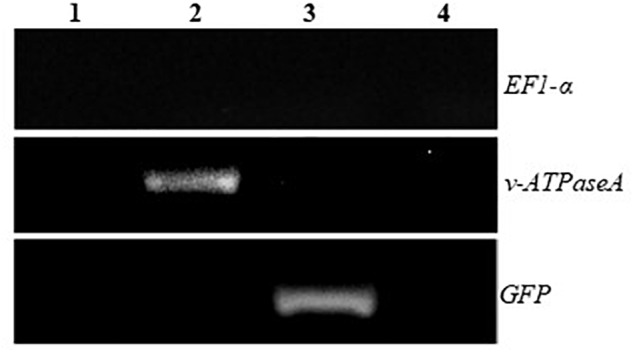
RT-PCR after removal of ssRNA using RNAseA to demonstrate the presence of annealed *v-ATPaseA* and *GFP* dsRNA in transplastomic plants. 1, WT plant; 2, *v-ATPAseA* dsRNA producing plant; 3, *GFP* dsRNA producing plant; and 4, No template control. Tobacco *EF1-α* gene specific primers were used to ensure that all ssRNA was removed by the RNAseA treatment.

Total RNA was extracted from the transplastomic plants and RT-PCR was performed using primers specific for the inserted sequences to check for expression of the dsRNA. Bands were present when transplastomic cDNA was used as the template, but not when WT cDNA was used, confirming that the plants express the desired products (Figure 3B). In addition, to confirm that the products transcribed from both strands of the DNA are annealed and present as dsRNA in the transplastomic plants, RT-PCR was performed using cDNA made from total RNA that was first digested with RNaseA to remove all ssRNA. PCR products of the expected sizes resulted when using *v-ATPAseA* and *GFP* primers with cDNA from *v-ATPAseA* and *GFP* dsRNA producing plants, respectively, confirming the presence of dsRNA products for these sequences (Figure 4). On the other hand, PCR performed using primers for a housekeeping gene in the plant, tobacco *EF1-α*, resulted in no products in any of the RNaseA-digested samples, confirming the efficient elimination of ssRNA from all the digested samples (Figure 4).

### Feeding on Transplastomic Plants Does Not Affect Insect Gene Expression or Survival

**FIGURE 5:**
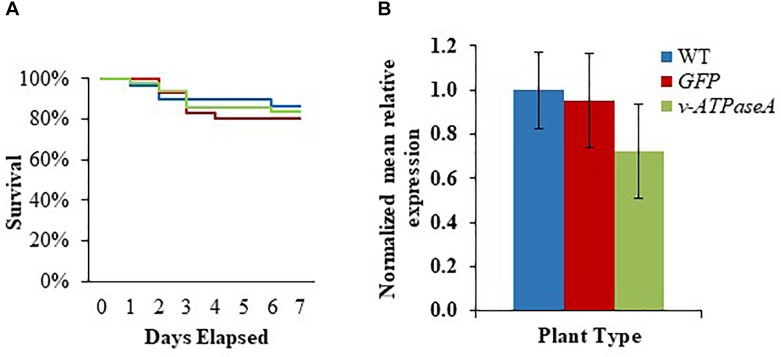
Survival of M. sexta first instar larvae and knockdown of *v-ATPaseA* gene after insects were fed either WT or transplastomic tobacco leaves expressing *v-ATPaseA* or *GFP* dsRNA over a 7-day period. **(A)** Kaplan–Meier survival analysis of insects over a 7-day period (*p* > 0.05, *n* = 44). **(B)** RT-qPCR showing relative expression of *v-ATPaseA* gene in insects after feeding on transplastomic and WT plants. Normalized mRNA quantity was set to one in WT tobacco-fed larvae and relative differences in mRNA levels in transplastomic tobacco-fed larvae were calculated. Data are expressed as mean relative quantity ± SEM (*p* > 0.05, *n* = 7 for *GFP, n* = 8 for *v-ATPaseA* and WT).

First instar larvae were placed on leaf disks from either WT, *v-ATPaseA* dsRNA-, or *GFP* dsRNA-producing tobacco plants and survival was observed over 7 days. After 7 days, mortality of insects feeding on *v-ATPaseA* dsRNA plants was 16.0%, compared to 20.0% for *GFP* dsRNA and 13.0% for WT tobacco (*p* ≥ 0.05) (Figure 5A). Furthermore, expression of *v-ATPaseA* mRNA was not significantly reduced (*p* ≥ 0.05) in insects that were fed transplastomic plants compared to insects fed WT plants (Figure 5B).

### Transplastomic Tobacco Plants Produce More Short Than Long dsRNA

**FIGURE 6:**
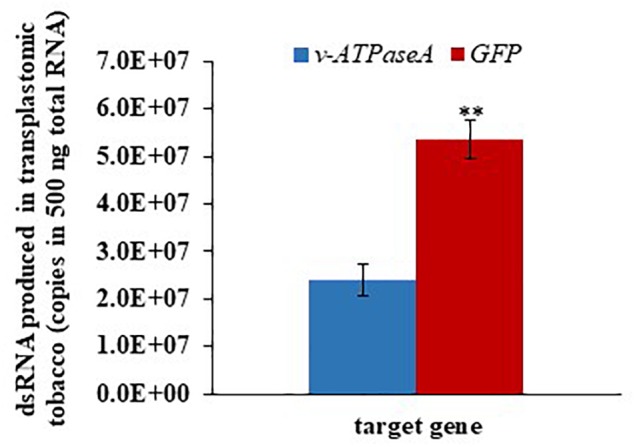
Absolute quantification of *v-ATPaseA* and *GFP* dsRNA in 500 ng transplastomic tobacco RNA. Data are expressed as mean quantity ± SEM. Asterisks represent significant changes in *t*-test (^∗∗^*p* ≤ 0.01), *n* = 3.

To determine whether the amount of dsRNA produced by transplastomic tobacco is affected by the dsRNA size, we quantified dsRNA levels using ddPCR. *GFP* dsRNA (223 bp) levels were significantly higher (*t* = 5.61; *p* = 0.00497, *n* = 3) than *v-ATPaseA* dsRNA (2222 bp) (Figure 6). In 500 ng total plant RNA, we estimated that there were approximately 2.39 × 10^7^ and 5.34 × 10^7^ dsRNA molecules in long *v-ATPaseA* and *GFP* dsRNA producing tobacco, respectively, assuming 100% conversion to cDNA. Overall, there was an increase of >two-fold in dsRNA molecules when the plants were transformed with the cassette producing the shorter *GFP* dsRNA compared to the cassette producing the longer *v-ATPase A* dsRNA.

## Discussion

Development of plants expressing high quantities of insecticidal dsRNA within their plastids is a promising strategy for future application in pest control. In this study, we showed that targeting of the *M. sexta v-ATPaseA* gene using an *in vitro* synthesized long dsRNA (2222 bp) induces higher mortality and gene knockdown compared to a short dsRNA (259 bp). Then, we transformed the plastid genome of tobacco with an expression cassette to produce the long dsRNA product and confirmed its production in the transplastomic plants. To our knowledge, this is the first reported instance of a dsRNA product this large being produced in a transplastomic plant. In addition, for the first time, our study compared the absolute quantities of dsRNA produced in plastids to determine whether length of the dsRNA has any effects on the amounts produced, which could be important for future applications using this approach to induce RNAi in insects.

Messenger RNAs encoding subunits of the v-ATPase protein complex are common targets for studies which aim to reduce survival of feeding pests by gene expression knockdown, including in *M. sexta* (Whyard et al., 2009). This protein complex has an important function in maintaining pH homeostasis and/or in membrane energization (Wieczorek et al., 2009); hence, interfering with expression of any subunit in the complex results in severe developmental and physiological defects and death (Baum et al., 2007; Badillo-Vargas et al., 2015; Jin et al., 2015; Li et al., 2015). Our observation, that a long *v-ATPaseA* dsRNA product generated through *in vitro* synthesis induces higher lethality and gene knockdown compared to a short *v-ATPaseA* dsRNA, is consistent with previously published studies (Bolognesi et al., 2012; Miller et al., 2012; Li et al., 2015). Although we did observe significantly greater mortality in short *v-ATPaseA* dsRNA-fed insects compared to water-fed insects, the results were not significantly different from *GFP* dsRNA-fed insects. Longer dsRNAs should yield larger populations of overlapping siRNA molecules ranging in size and sequence (Nandety et al., 2015), and this would be expected to improve RNAi efficiency in comparison to shorter dsRNA products. Also, variation in RNAi efficiency due to different dsRNA lengths has been attributed to less efficient recognition of shorter dsRNA by cellular uptake mechanisms (Saleh et al., 2006). Both of these factors might contribute to a bias toward greater RNAi efficiency of longer dsRNAs.

Because we observed higher mortality with the long dsRNA, we transformed tobacco plants to produce the long dsRNA product within their plastids. We confirmed that annealed double-stranded products are present in the transplastomic plants using RT-PCR. Removal of ssRNA before cDNA synthesis provided evidence that dsRNA molecules are formed in the transplastomic plants after transcription. Previously, it was also shown that dsRNA produced in plastids accumulated stably, and was not processed into siRNA molecules, as there is no dsRNA-processing machinery present in these subcellular compartments (Zhang et al., 2015). However, despite the effectiveness of the droplet feeding approach, insects feeding upon transplastomic plants producing the long dsRNA neither showed a significant increase in mortality nor a drop in *v-ATPaseA* transcript level from controls, although the levels observed were suggestive of a trend to lower expression. There are several possible explanations for why the *in planta* approach failed to produce an effective dose of dsRNA. The most likely is that the *in planta*-produced dsRNA is simply present in lower quantities than those attained by feeding of *in vitro*-synthesized dsRNA. RNAi response can be dependent on dsRNA concentrations (Kumar et al., 2009; Tian et al., 2009), and in particular, high doses of dsRNA might be required when dsRNA is delivered orally (Luo et al., 2013; Scott et al., 2013).

When we compared the absolute quantities of dsRNA produced in plants transformed with the long *v-ATPaseA* gene (2222 bp) with those of the control plants producing the shorter *GFP* gene fragment (223 bp), we found that accumulation of the long dsRNA was lower by more than two-fold than that of the shorter dsRNA. This implies that expression of a very long product *in planta* may be a limiting factor in achieving high concentrations of dsRNA. One potential influence on the yield of dsRNA in our transplastomic plants stems from the efficiency with which such products would be synthesized. The strong chloroplast promoter, *Prrn*, used in our study to drive dsRNA expression was previously shown to result in high mRNA accumulation levels which led to exhaustion of the plastid translational machinery (Oey et al., 2009). However, the stability of the RNA transcripts produced will affect the final levels achieved. In fact, it was previously suggested that stability of RNA molecules plays an important role in RNA quantities in plastids (Mullet, 1993), and the sequence of the dsRNA may influence its stability (Zhang et al., 2015). The stability of RNA molecules can also be influenced by their length, as longer transcripts have been shown to be more prone to degradation by nucleases as well as mechanical damage compared to their shorter counterparts (Feng and Niu, 2007). Furthermore, it is also possible that long nascent RNA sequences could form secondary structures under cellular conditions (Sükösd et al., 2013), which could prevent stable dsRNA annealing, and cause more rapid polymerase dissociation from the template. It has been suggested that polymerase enzymes transcribing longer dsRNA products can dissociate from the template more often, which would result in truncated products prone to rapid degradation (Cagliero and Jin, 2013).

Another potential factor influencing the effective dose of dsRNA to a feeding insect is the presence of nucleases that degrade dsRNA. The importance of dsRNA degrading nucleases in RNAi efficiency using orally delivered dsRNA was highlighted by a study that demonstrated improved RNAi response upon silencing of nucleases in the Colorado potato beetle midgut (Spit et al., 2017), an insect known to be sensitive to RNAi (Palli, 2014). The effect of nucleases on RNAi efficiency is dependent on the species involved (Joga et al., 2016; Garcia et al., 2017; Guan et al., 2018), and several studies have demonstrated that dsRNA molecules are degraded faster in the midgut and hemolymph of lepidopteran insects compared to other Orders (Garbutt et al., 2013; Wang et al., 2016; Singh et al., 2017). When we fed *in vitro* synthesized dsRNA to *M. sexta* larvae, we first starved the insects for 4–6 h; whereas, the plant fed larvae were not starved and were provided with leaves continuously to deliver the dsRNA. Previously, it has been shown that starvation can influence the midgut environment and reduce the nuclease activities in the insect, resulting in increased dsRNA stability (Rodríguez-Cabrera et al., 2010; Cooper et al., 2018). This difference presents another potential explanation for the lower RNAi effectiveness we observed using *in planta* delivered dsRNA. Additionally, a lepidopteran-specific nuclease has been shown to be upregulated in response to dsRNA exposure in another lepidopteran insect, Asian corn borer (Guan et al., 2018). Continuous feeding by the larvae on dsRNA-producing plants may result in detection of low quantities of dsRNA early by the midgut cells, resulting in upregulation of nucleases to degrade the dsRNA molecules. Therefore, slow consumption of the dsRNA *in planta* over a longer period of time may produce both a reduced dsRNA dose level compared to a one-time ingestion of high quantities of dsRNA by starved insects, as well as lead to greater presence of nucleases in the insect gut.

Taken together, our results suggest that lower amounts of long dsRNA produced in our transplastomic plants were insufficient, in comparison with *in vitro* produced material fed as a bolus, to induce RNAi in *M. sexta* larvae. It is possible that the level of dsRNA produced in our transplastomic plants would be sufficient to induce lethality in insects that are more susceptible to dsRNA, and representatives of other Orders are worth investigating in the future. However, in general, the results of this study suggest that a size-dependent limit on high level dsRNA production in plastids exists, and future research needs to be directed to determining the point at which the benefits of more efficient long dsRNA uptake are balanced with the decline in production efficiency for long dsRNA products in the plastid.

## Data Availability

All datasets generated for this study are included in the manuscript and/or the Supplementary Files.

## Author Contributions

WB, CD, and RM contributed conception and design of the study. WB and EK performed the experiments. CD, IK, and RM contributed reagents, materials, and analysis tools. WB wrote the first draft of the manuscript. EK, IK, and CD wrote sections of the manuscript. All authors contributed to manuscript revision, read, and approved the submitted version.

## Conflict of Interest Statement

The authors declare that the research was conducted in the absence of any commercial or financial relationships that could be construed as a potential conflict of interest.
