# Supplementary material for: RNA Interference in the Tobacco Hornworm, Manduca sexta, Using Plastid-Encoded Long Double-Stranded RNA
Source: Front Plant Sci. 2019 Mar 14;10:313. doi: 10.3389/fpls.2019.00313 (PMC6426776; doi:10.3389/fpls.2019.00313)
Supplement: Supplementary file 1 [file Data_Sheet_1.pdf]

Supplementary Materials

RNA interference in the tobacco hornworm, *Manduca sexta*, using plastid-encoded long double-stranded RNA

William G. Burke, Emine Kaplanoglu, Igor Kolotilin, Rima Menassa, Cam Donly*

*** Correspondence:**
Cam Donly
cam.donly@canada.ca

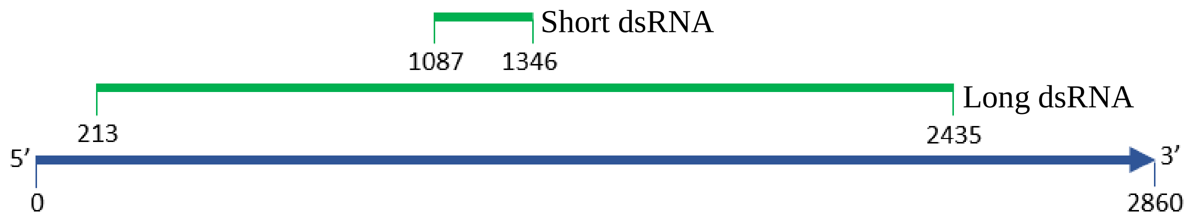

Supplementary Figure 1. Coverage of double-stranded RNA regions compared to full-length *Manduca sexta v-ATPaseA* mRNA (GenBank: X64233.1). Values indicate the number of nucleotides from 5' end of mRNA.

Supplementary Figure 2. Sequence alignment of *M. sexta v-ATPaseA* gene with human and tobacco homologues (aligned using MUSCLE in MEGA 7).

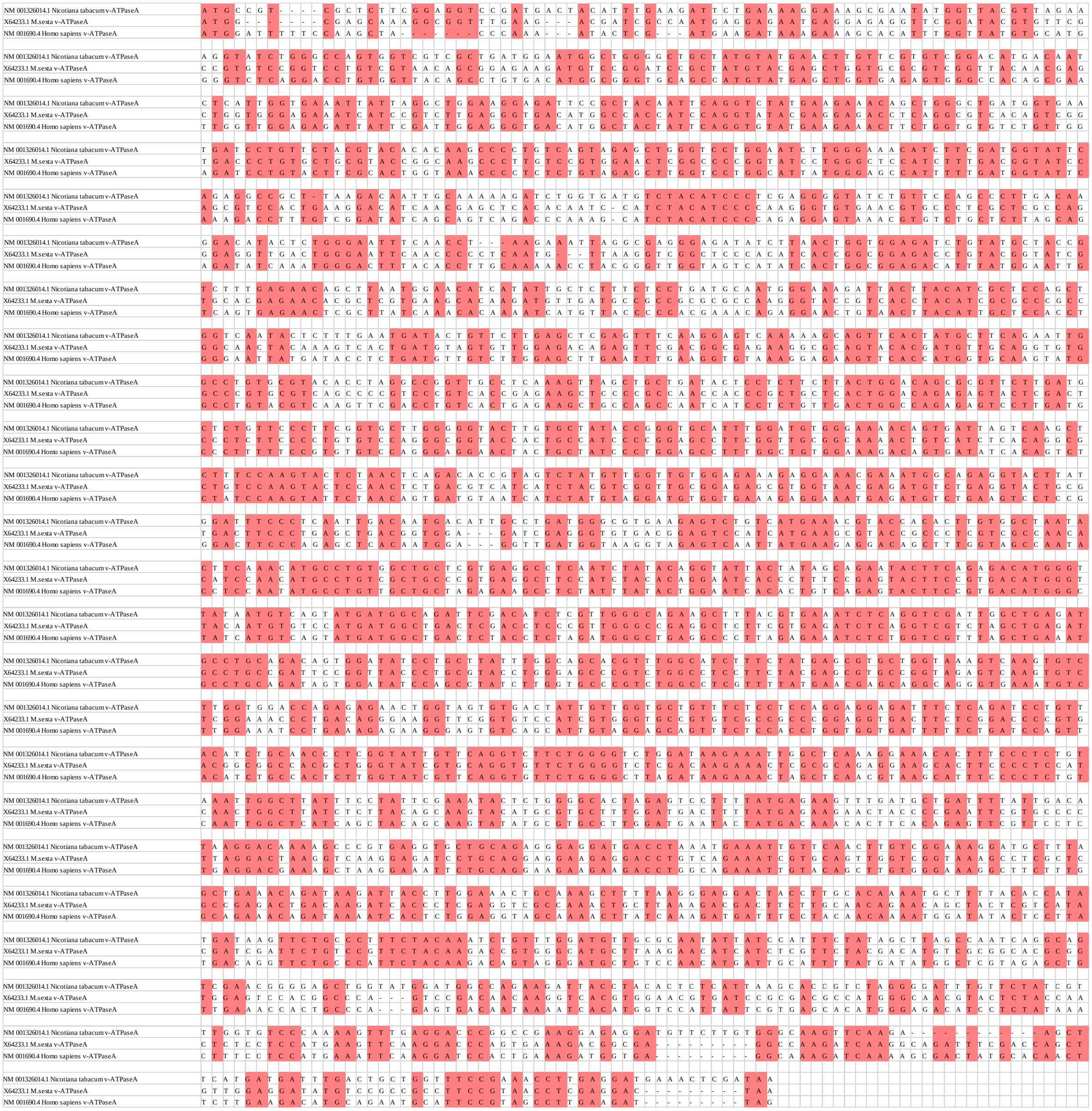

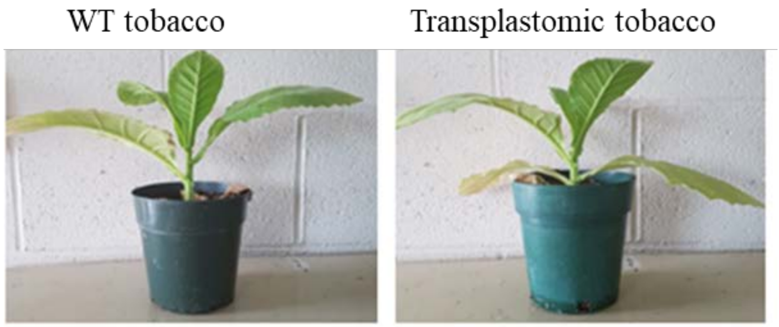

Supplementary Figure 3. Phenotype of WT tobacco versus *v-ATPaseA* dsRNA-producing tobacco. No differences in phenotype were observed.

Supplementary Table 1: Oligonucleotides used in this study.

Primer name	Sequence (5'-3') (square brackets indicate T7 promoter sequence or restriction enzyme cut sites)
**Primers used for *in vitro* dsRNA synthesis**	
v-ATPaseA-L-T7 Fwd	[TAATACGACTCACTATAGGG]AATCATCCGTCTTGAGGGTGA
v-ATPaseA-L-T7 Rev	[TAATACGACTCACTATAGGG]CCACTGTGACGACTGTGTT
v-ATPaseA-S-T7 Fwd	[TAATACGACTCACTATAGGG]ATGATGGCTGACTCGACCT
v-ATPaseA-S-T7 Rev	[TAATACGACTCACTATAGGG]ACACCTGCACGATACCCAG
GFP-T7 Fwd	[TAATACGACTCACTATAGGG]ACTTTTCACTGGAGTTGT CCCAA
GFP-T7 Rev	TAATACGACTCACTATAGGG]AGTAGTGACAAGTGTTGGCTGA
**Primers used for cloning of dsRNA sequences into pTomCT vector**	
*Not*I-*v-ATPaseA*-L Fwd	[GCGGCCGC]AATCATCCGTCTTGAGGGTGA
*Sal*I-*v-ATPaseA*-L Rev	[GTCGAC]CCACTGTGACGACTGTGTT
*Not*I-*GFP* Fwd	[GCGGCCGC]ACTTTTCACTGGAGTTGTCCCAA
*Sal*I-*GFP* Rev	[GTCGAC]AGTAGTGACAAGTGTTGGCTGA
**Primers used for confirmation of insertion/expression**	
v-ATPaseA Fwd	ATGATGGCTGACTCGACCT
v-ATPaseA Rev	ACACCTGCACGATACCCAG
GFP Fwd	ACTTTTCACTGGAGTTGTCCCAA
GFP Rev	AGTAGTGACAAGTGTTGGCTGA
NtEF1-α Fwd	TTGGTCGTGTGGAAACTGG
NtEF1-α Rev	CCAACATTGTCACCAGGAAGTG
**Primers used for DIG-labelled probe synthesis**	
DP*Nt* Fwd	TCAATCCCTTTGCCCCTCAT
DP*Nt* Rev	TCAACTGCCCCTATCGGAA
**Primers used for RT-qPCR of insect RNA**	
qMsv-ATPaseA Fwd	CAAAGGGCGTGGCTTGTAAA
qMsv-ATPaseA Rev	ACCAACCCAGGTCCAAACAT
qMsEF1-α Fwd	GTGTCCGTCAAGGAATTGCG
qMsEF1-α Rev	TCCAGGGTGGTTGAGTACGA

Supplementary Table 1 Cont: Oligonucleotides used in this study.

**Primers and probes used for ddPCR**	
NtRPL25 For	AATCTGACCCCAAGGCACAG
NtRPL25 Rev	AGTCTTAGGTCGGTGGAATGT
NtRPL25 Probe	[6~FAM]CCAAGGCTGTCAAGTCAGGATCAACC[BHQ1a~Q]
GFP For	CGTATGTTGCATCACCTTCA
GFP Rev	TGGAGTTGTCCCAATTCTTG
GFP Probe	[6~FAM]CCCTCTCCACTGACAGAAAATTTGTGCC[BHQ1a~Q]
v-ATPaseA For	GAGGCTCTTCGTGAGATC
v-ATPaseA Rev	CCGAGACACTTGACTCTAC
v-ATPaseA Probe	[6~FAM]TCGTCTAGCTGAGATGCCTGCCG[BHQ1a~Q]

Supplementary Table 2. Double-stranded RNA sequences used in this study.

**Sequence description**	**Sequence**
***v-ATPAseA* long**	AATCATCCGTCTTGAGGGTGACATGGCCACCATCCAGGTAT ACGAGGAGACCTCAGGCGTCACAGTCGGTGACCCTGTGCTG CGTACCGGCAAGCCCTTGTCCGTGGAACTCGGCCCCGGTAT CCTGGGCTCCATCTTTGACGGTATCCAGCGTCCACTGAAGG ACATCAACGAGCTCACACAATCCATCTACATCCCCAAGGGT GTGAACGTGCCCTCGCTCGCCAGGGAGGTTGACTGGGAATT CAACCCCCTCAATGTTAAGGTCGGCTCCCACATCACCGGCG GAGACCTGTACGGTATCGTGCACGAGAACACGCTCGTGAAG CACAAGATGTTGATGCCGCCGCGCGCCAAGGGTACCGTCAC CTACATCGCGCCCGCCGGCAACTACAAAGTCACTGATGTAG TGTTGGAGACAGAGTTCGACGGCGAGAAGGCGCAGTACAC GATGTTGCAGGTGTGGCCCGTGCGTCAGCCCCGTCCCGTCA CCGAGAAGCTCCCCGCCAACCACCCGCTGCTCACTGGACAG AGAGTACTCGACTCCCTCTTCCCCTGTGTCCAGGGCGGTACC ACTGCCATCCCCGGAGCCTTCGGTTGCGGCAAAACTGTCAT CTCACAGGCGCTGTCCAAGTACTCCAACTCTGACGTCATCAT CTACGTCGGTTGCGGAGAGCGTGGTAACGAGATGTCTGAGG TACTGCGTGACTTCCCTGAGCTGACGGTGGAGATCGAGGGT GTGACGGAGTCCATCATGAAGCGTACCGCCCTCGTCGCCAA CACATCCAACATGCCTGTCGCTGCCCGTGAGGCTTCCATCTA CACAGGAATCACCCTTTCCGAGTACTTCCGTGACATGGGTT ACAATGTGTCCATGATGGCTGACTCGACCTCCCGTTGGGCC GAGGCTCTTCGTGAGATCTCAGGTCGTCTAGCTGAGATGCC TGCCGATTCCGGTTACCCTGCGTACCTGGGAGCCCGTCTGGC CTCCTTCTACGAGCGTGCCGGTAGAGTCAAGTGTCTCGGAA ACCCTGACAGGGAAGGTTCGGTGTCCATCGTGGGTGCCGTG TCGCCGCCCGGAGGTGACTTCTCGGACCCCGTGACGGCGGC

CACGCTGGGTATCGTGCAGGTGTTCTGGGGTCTCGACAAGA
AACTCGCGCAGAGGAAGCACTTCCCCTCCATCAACTGGCTT
ATCTCTTACAGCAAGTACATGCGTGCTTTGGATGACTTTTAT
GAGAAGAACTACCCCGAATTCGTGCCCCTTAGGACTAAGGT
CAAGGAGATCCTGCAGGAGGAAGAGGACCTGTCAGAAATC
GTGCAGTTGGTCGGTAAAGCCTCGCTCGCCGAGACTGACAA
GATCACCCTCGAGGTCGCCAAACTGCTTAAAGACGACTTCT
TGCAACAGAACAGCTACTCGTCATACGATCGATTCTGTCCG
TTCTACAAGACCGTGGGCATGCTTAAGAACATCATCTCGTTC
TACGACATGTCGCGGCACGCGGTGGAGTCCACGGCCCAGTC
CGACAACAAGGTCACGTGGAACGTGATCCGCGACGCCATGG
GCAACGTACTCTACCAACTCTCCTCCATGAAGTTCAAGGAC
CCAGTGAAAGACGGCGAGGCCAAGATCAAGGCAGATTTCG
ACCAGCTGTTGGAGGATATGTCCGCCGCCTTCCGTAACCTC
GAGGACTAAGCACAGCCGTACTACAGTACAGTACAGTAGG
GAGCGCCCACGAGCCGCGCCGCGACATCCTCCGCAGCCGAG
AGGACATCTTTATCGACTTGTTTTCATGTTGTCATTTTTATTA
TAATTTATTGATTAATATGAGGATATATTTTTTCGTATTCTAT
TCACGTCCGGAGCGTTTTGAGACAGTTTTTTCGAGTCTGGAG
TGTTTTGCATTTTATCGATATTATCGAGTGTCGGGCGTCGTT
AAGGCGGTGCTGTTAGCGAGGTATGCGTTATGACACACGCA
TATATCGTAATAACAGCGTTGTTTAAACGGGTCTGTGCGCA
GGCGCAGTTCGTGGGCGGTCGTGTTGTTATAGTAATTATGTA
GTGTTAAATATATTACAACATCGATTCCAGAGGATGGTGTC
GCGGGCTAGAACTCCGACAGCGCGAAAGCCTACAAAGGGC
GTGGCTTGTAAACGGCACAATAAGGCCGACTAACAATTCTC
CGTTATTTGAAATAGCAGTTCAAACACAGTCGTCACAGTGG

***v-ATPaseA* short**

ATGATGGCTGACTCGACCTCCCGTTGGGCCGAGGCTCTTCG
TGAGATCTCAGGTCGTCTAGCTGAGATGCCTGCCGATTCCG
GTTACCCTGCGTACCTGGGAGCCCGTCTGGCCTCCTTCTACG
AGCGTGCCGGTAGAGTCAAGTGTCTCGGAAACCCTGACAGG
GAAGGTTCGGTGTCCATCGTGGGTGCCGTGTCGCCGCCCGG
AGGTGACTTCTCGGACCCCGTGACGGCGGCCACGCTGGGTA
TCGTGCAGGTGT

GFP

ACTTTTCACTGGAGTTGTCCCAATTCTTGTTGAATTAGATGG
TGATGTTAATGGGCACAAATTTTCTGTCAGTGGAGAGGGTG
AAGGTGATGCAACATACGGAAAACTTACCCTTAAATTTATT
TGCACTACTGGAAAACTACCTGTTCCATGGGTAAGTTTAAA
CATATATATACTAACTAACCCTGATTATTTAAATTTTCAGCC
AACACTTGTCACTACT
